# Novel Insights into the Immunotherapy of Soft Tissue Sarcomas: Do We Need a Change of Perspective?

**DOI:** 10.3390/biomedicines9080935

**Published:** 2021-08-01

**Authors:** Andrej Ozaniak, Jiri Vachtenheim, Robert Lischke, Jirina Bartunkova, Zuzana Strizova

**Affiliations:** 1 Third Department of Surgery, First Faculty of Medicine, Charles University and University Hospital Motol, 150 06 Prague, Czech Republic; andrej.ozaniak@fnmotol.cz (A.O.); jiri.vachtenheim@fnmotol.cz (J.V.J.); robert.lischke@fnmotol.cz (R.L.); 2 Department of Immunology, Second Faculty of Medicine, Charles University and University Hospital Motol, 150 06 Prague, Czech Republic; jirina.bartunkova@fnmotol.cz

**Keywords:** sarcoma, immunotherapy, checkpoint inhibitors, adoptive transfer, trabectedin, IL-15, tumor microenvironment, TILs, immune cells

## Abstract

Soft tissue sarcomas (STSs) are rare mesenchymal tumors. With more than 80 histological subtypes of STSs, data regarding novel biomarkers of strong prognostic and therapeutic value are very limited. To date, the most important prognostic factor is the tumor grade, and approximately 50% of patients that are diagnosed with high-grade STSs die of metastatic disease within five years. Systemic chemotherapy represents the mainstay of metastatic STSs treatment for decades but induces response in only 15–35% of the patients, irrespective of the histological subtype. In the era of immunotherapy, deciphering the immune cell signatures within the STSs tumors may discriminate immunotherapy responders from non-responders and different immunotherapeutic approaches could be combined based on the predominant cell subpopulations infiltrating the STS tumors. Furthermore, understanding the immune diversity of the STS tumor microenvironment (TME) in different histological subtypes may provide a rationale for stratifying patients according to the TME immune parameters. In this review, we introduce the most important immune cell types infiltrating the STSs tumors and discuss different immunotherapies, as well as promising clinical trials, that would target these immune cells to enhance the antitumor immune responses and improve the prognosis of metastatic STSs patients.

## 1. Introduction

Soft tissue sarcomas (STSs) are a heterogeneous group of rare tumors arising from mesenchymal tissues [1]. STSs can originate from any human body location and, with more than 80 histological subtypes of STSs, data regarding novel biomarkers of strong prognostic and therapeutic value are very limited [2,3]. The most prevalent histological subtypes of STSs include liposarcoma, undifferentiated pleomorphic sarcoma, and leiomyosarcoma [4]. Most STS occur spontaneously and present as an asymptomatic soft tissue mass [5,6]. Nevertheless, certain factors, such as exposure to radiation and chemicals or genetic aberrations, were also previously associated with the risk of developing STSs [6].

The American Joint Committee on Cancer (AJCC) staging system for STSs relies on the histologic grade, the tumor size and depth, and the presence of distant or nodal metastases [7,8,9]. To date, the most important prognostic factor is the tumor grade [9]. Approximately 50% of patients that are diagnosed with high-grade STSs die of metastatic disease [10]. Most STSs are known for early hematogenous metastasizing [11]. The disease rarely affects the lymphatic system, but the impairment of the lymph nodes is a sign of high tumor aggressiveness [12,13]. The predominant site of metastases are the lungs. However, retroperitoneal STSs also tend to metastasize to the liver [14,15]. Other metastatic sites commonly include the bones and the brain [16,17].

Patients with STSs are managed according to the generally accepted guidelines and those with localized and resectable diseases are treated by surgery [18]. The mainstay of STS treatment is a complete surgical resection of the tumor with ensured negative margins [19]. Although major improvements in the local control rates are achieved, the success of surgery critically depends on the tumor location, tumor size, the involvement of nearby structures, and other factors [19,20]. With optimally surgically treated localized disease, approximately 50% of high-grade STS patients eventually develop pulmonary metastases within five years [2,21,22,23]. Both neoadjuvant and adjuvant radiotherapy have reduced the local recurrences but were also associated with significant toxicity, especially in retroperitoneal STSs [24,25].

Metastatic STSs have very limited treatment options [26]. Systemic chemotherapeutic agents induce response in only 15–35% of the patients, irrespective of the histological subtype [27,28,29]. Doxorubicin represents the mainstay of treatment for decades and only small benefit was observed when combined with other chemotherapeutic agents [27,28,29]. The median survival of STSs patients after the administration of by chemotherapy is only 10–15 months [27,28,29].

Cancer immunotherapy has changed the treatment landscape in oncology, modified the therapeutic algorithms in multiple malignancies and, furthermore, became the leading treatment for metastatic diseases [30]. With the diverse immunotherapeutic approaches that are currently being applied, complete remissions have been observed in some patients [31,32,33]. However, a significant proportion of patients are immunotherapy resistant [34]. STSs belong to the tumors with only limited responses to immunotherapy [35,36].

While molecular characteristics of STSs are being urgently investigated among studies, the understanding of the events that occur within the tumor-immune system interplay in STSs are far from satisfactory [37]. The phenotypic profile of immune infiltrates of STSs should drive the process of decision-making whether to apply immunotherapy [34,38,39,40]. Furthermore, deciphering the immune cell signatures within the tumor may discriminate immunotherapy responders from non-responders and different immunotherapeutic approaches could be combined based on predominant cell subpopulations infiltrating the STS tumors [34,39,40].

In this review, we introduce the most important immune cell types infiltrating the STSs tumors and discuss different immunotherapies that would target these immune cells to enhance the anti-tumor immune responses and improve the prognosis of metastatic STSs patients.

## 2. Methods

**Figure 1:**
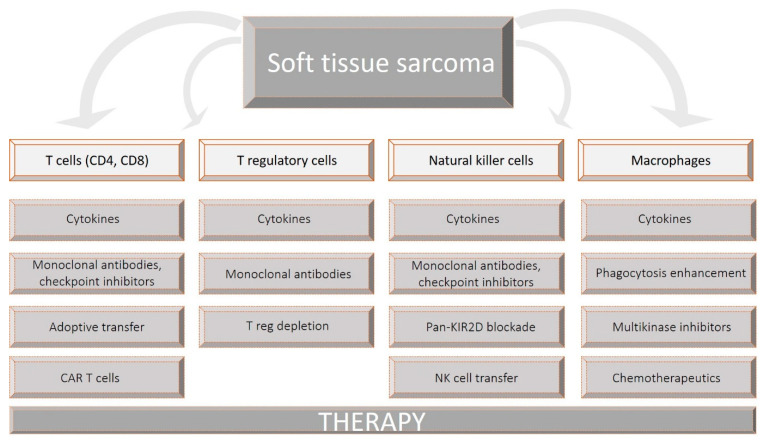
Scheme of the data selection process. Soft tissue sarcoma, T cells, CD3 T cells, CD8 T (cytotoxic) cells, CD4 T (helper) cells, natural killer (NK) cells, macrophages, T regulatory cells, Tregs, and FOXP3 T cells were used as the key words in the search strategy. Boxes below cell populations represent the search sub-categories. Therapies targeting the particular immune cell population were searched through the official registry at clinicaltrials.gov and search databases Medline/Pubmed, Scopus, and Web of Science. Articles published within the past ten years (Jan 1st 2011–July 1st 2021) were primarily taken into consideration.

A comprehensive review of the literature on diverse immune cell populations infiltrating the tumor microenvironment (TME) of STSs and a review of therapeutic approaches targeting these cell populations was conducted. Soft tissue sarcoma, T cells, CD8 T (cytotoxic) cells, CD4 T (helper) cells, natural killer (NK) cells, macrophages, T regulatory cells, Tregs, and FOXP3 T cells were used as the key words in the search strategy. The diagnosis of osteosarcoma, as well as Kaposi sarcoma, was excluded. Therapies targeting the particular immune cell population were searched through the official registry at clinicaltrials.gov and search databases. Excluded were clinical trials of unknown status and withdrawn clinical trials. Only English written and peer-reviewed articles published in indexed international journals until June 2021 were reviewed. Databases used for search included Medline/Pubmed, Scopus, and Web of Science. The selection process is summarized in Figure 1.

## 3. T Cell Infiltration

T helper cells (CD4^+^) and cytotoxic T cells (CD8^+^) are the two main subpopulations of T cells that control and shape the immune responses in the tumor microenvironment (TME) [41].

T cells when activated through their T cell receptor (TCR) serve as the mediators of the adaptive immune response that efficiently migrate into the TME and induce cellular death in their target tumor cells [41,42]. The predominant cytotoxic mechanisms of CD8^+^ T cells include the production of death receptor ligands, such as Fas ligand or TRAIL; the production of perforin and granzyme; and the production of cytokines, such as TNF [43]. In most cancer types, the expression of immune checkpoint receptors, such as PD-1, CTLA-4, TIM-3, Lag-3, and other inhibitory molecules in CD8^+^ T cells is associated with the disease prognosis and is highly predictive of efficient immunotherapy with immune checkpoint inhibitors (CPIs) [44].

CD4^+^ T cells in the TME primarily serve as promotors of the executive functions of effector CD8^+^ T cells [45]. However, CD4^+^ T cells were also shown to bear cytolytic abilities and were proposed by a number of studies to represent the major anti-tumor T cell subpopulation [46]. CD4^+^ T cells differentiate into multiple cell sublineages, out of which Th1 cells are probably the most potent Th lineage against tumors [45].

**Figure 2:**
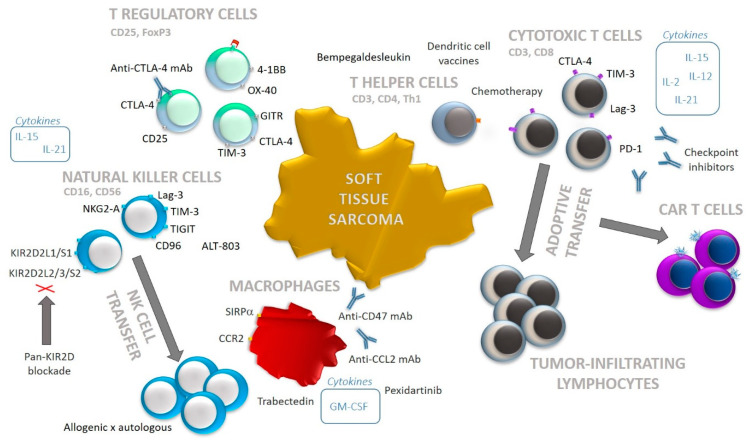
A schematic diagram for major cell populations infiltrating the tumor microenvironment of STSs and an overview of different therapies targeting these populations. Surface receptors can be targeted with monoclonal antibodies (mAbs), and diverse cytokines serve as potent promotors of cell functions. CD3^+^ T cells and natural killer (NK) cells can be ex vivo expanded and adoptively transferred to a patient. Moreover, a generation of chimeric antigen receptor (CAR) T cells is allowed by a genetic modification of T cell receptor (TCR). Protumorigenic (M2) macrophages, as well as T regulatory cells, can be selectively depleted by synthetic agents or mAbs. The phagocytic capacity of macrophages, on the other hand, is secured by an administration of anti-CD47 mAbs. NK cells can be efficiently activated by a pan-KIR2D blockade together with IL-15 superagonist. Multiple immunotherapeutic approaches can be combined.

Both cell populations, CD8^+^ and CD4^+^ T cells, also regulate the immune responses in the TME by secreting a broad range of cytokines Figure 2 [47]. 

Different studies attempted to assess the proportions and phenotypes of T cells in STSs [48,49]. A recent study by Klaver et al. demonstrated that nearly one third of liposarcomas and leiomyosarcomas belong to the CD8^+^ T cell-poor tumors, whereas pleomorphic sarcoma and myxofibrosarcoma were shown to have one of the highest infiltration with CD8^+^ T cells [48]. Conversely, a study by Pollack et al. suggested that leiomyosarcomas belong to the inflammatory tumor types with high levels of T-cell-related gene expression, with several tumors demonstrating expression of PD-1 and very strong expression of PD-L1 [49]. However, studies evaluating the efficacy of anti-PD1 agents in the treatment of leiomyosarcomas were greatly disappointing [49]. Klaver et al. presented that only 7% of myxofibrosarcomas and 13% of pleomorphic sarcomas were poorly infiltrated with CD8^+^ T cells [48]. Moreover, pleomorphic sarcomas and myxofibrosarcomas had also the highest fractions of PD-1^+^CD8^+^ T cells [48]. Interestingly, pleomorphic sarcoma displayed highest proportions of PD-1^+^Lag-3^+^TIM-3^+^CD8^+^ TILs, being comparable to malignant melanoma [48]. These results are also supported by a recent pooled analysis of anti-PD1 and anti-PD-L1 phase II clinical trials where undifferentiated pleomorphic sarcoma exhibited the highest response rates to treatment [50]. Another study by D’Angelo et al. showed that leiomyosarcoma, liposarcoma, synovial sarcoma and chondrosarcoma generally had low-density CD8^+^ cells [51]. In another study, synovial sarcoma had significantly increased concentrations of CD8^+^ TILs expressing PD-1 in metastatic tumors as compared to primary tumors [52].

Previous studies have discussed the association between the clinicopathologic factors and the infiltration of the TME with TILs [53,54,55,56]. It was also previously shown that one of the main challenges of successful immunotherapy is the inefficient T cell trafficking into the tumor tissue [34,57]. A recent study by Wustrack et al. has shown that in undifferentiated pleomorphic sarcoma, larger tumors limited the immune infiltration with CD8^+^ T cells as the tumor size significantly correlated with a decrease in the frequency of CD8^+^ TILs [58]. Also, a high load of effector CD8^+^ T cells was associated with improved overall survival in these STSs [58]. According to the TME immunoprofiling among several different studies, undifferentiated pleomorphic sarcomas generally have a potential to respond to anti-PD-1 immunotherapy [35,58].

The assessment of PD-1 and PD-L1 expression status among STSs has been carried out by Movva et al. [59]. The report of 2000 sarcomas revealed over 50% of PD-1 and PD-L1 positive TILs in the TME of sarcomas [59]. However, both STSs and bone sarcomas were included into the study [59]. The PD-L1 expression was observed in 70% of undifferentiated pleomorphic sarcoma cases, in 75% of chondrosarcoma cases, in 77% of liposarcomas cases, but only in 32% of leiomyosarcomas cases [59].

Data regarding the proportions and roles of CD4^+^ T cells in the STS TME are limited and only little is known about their phenotypic patterns. Recently, authors Bi et al. have shown that the infiltration with CD4^+^ T cells positively correlated with better survival in STSs and could, thus, serve as a prognostic biomarker for STSs [60]. Moreover, in this study, CD4^+^ T cell infiltration levels were significantly associated to the overall survival in patients with undifferentiated pleomorphic sarcomas [60]. Another recent study showed the CD4^+^ T cell expression in leiomyosarcomas approximately 30% (+/−22%) but contained a large proportion of T regulatory cells (Tregs) while lacking the activation markers, such as CD69 and CD32 [61].

## 4. T Cell Immunotherapies in Soft Tissue Sarcomas

An immunotherapeutic approach that relies on the administration of stimulatory cytokines serves as an important regulator of T cell functions [62]. To date, the most commonly applied cytokine remains the interleukin-2 (IL-2) Figure 2 [62]. However, a careful balance must be achieved when selecting the optimal IL-2 concentration to avoid a preferential induction of CD4^+^CD25^+^Foxp3^+^ T regulatory cell (Treg) expansion [62]. Other cytokines, such as IL-12, IL-15, IL-21, and granulocyte macrophage colony-stimulating factor (GM-CSF), are still being evaluated in clinical trials [62].

In STSs, over 20 clinical trials have been initiated with the administration of IL-2 in a combination therapy. However, none of these trials have entered phase III of clinical testing (clinicaltrials.gov). A single-arm multi-cohort phase II study is currently ongoing with the aim to evaluate the immunological effectiveness and safety of IL-2 in combination with autologous dendritic cell vaccination (NCT04166006). Both approaches, IL-2 and DC vaccination should provide stimulatory signals to T cells and thus, promote the adaptive T-cell mediated immune responses in the TME [63,64]. Another phase II clinical trial in STSs is based on the administration of IL-2 in combination with autologous TILs and chemotherapy (NCT03449108) and the results are expected in 2022. A total of four clinical trials have been initiated with the IL-15, a potent activator of NK cells and T cells, but not Tregs [65]. Out of these phase I clinical trials, one trial is based on the administration of autologous activated T-cells expressing a second generation GD2 Chimeric Antigen Receptor (CAR), IL-15 and iCaspase9, and is currently recruiting (NCT03721068).

CPIs are blocking monoclonal antibodies (mAbs) targeting surface inhibitory receptors of T cells (Figure 2) [66]. To date, the most compelling results in clinical practice were observed with anti-CTLA-4 mAbs (ipilimumab, tremelimumab), anti-PD-1 mAbs (nivolumab, pembrolizumab, pidilizumab), and anti-PD-1-ligand mAbs (atezolizumab, avelumab, durvalumab) [67]. The efficacy of anti-PD-1 depends on its ability to restrain the TCR signaling pathway [68]. Anti-CTLA-4, on the other hand, is a competitive receptor that prevents binding of CD80/86 [69].

Response to immunotherapy with CPIs largely depends on the level of CD8^+^ TILs infiltration in the TME and on the expression of immune checkpoint molecules [34]. Since STSs are infiltrated with TILs in diverse proportions, many clinical trials with anti-PD-1, anti-PD-L1 and anti-CTLA4 have been initiated to distinguish the responders from non-responders. To date, 48 clinical trials have been initiated in STSs patients with nivolumab (anti-PD-1), 39 with pembrolizumab (anti-PD-1), 30 with ipilimumab (anti-CTLA-4), 13 with atezolizumab (anti-PD-L1), 17 with durvalumab (anti-PD-L1), 9 with avelumab (anti-PD-L1), 5 with tremelimumab (anti CTLA-4), and 2 with cemiplimab (anti-PD-1) (clinicaltrials.gov). Only one CPI study has reached phase III of clinical testing and is currently recruiting (NCT04741438). In this French randomized prospective multicentre study, STSs patients will receive a combination of nivolumab (3 mg/kg) and ipilimumab (1 mg/kg) for a maximum treatment period 12 months (NCT04741438). The inclusion criteria cover patients with metastatic or unresectable advanced sarcomas of rare subtype.

Out of the most commonly applied CPIs, nivolumab, pembrolizumab, and ipilimumab entered phase II clinical trials with a great deal of promise (Table 1).

In an observational phase II clinical trial, pembrolizumab was administered in monotherapy to 42 STSs patients with diverse tumor histology (NCT02301039). In this study, one complete response was observed in a patient with undifferentiated pleomorphic sarcoma and a total of seven patients experienced objective responses [70]. To note, not a single leiomyosarcoma patient responded to the treatment with pembrolizumab [70]. Combination therapies with pembrolizumab include active and/or recruiting clinical trials with chemotherapy (NCT03123276), radiotherapy (NCT03338959), biologic therapy (NCT03126591), and tyrosine kinase inhibitors (NCT02636725).

Nivolumab is currently being evaluated for the treatment of STSs mostly in combination therapies (clinicaltrial.gov). Nivolumab monotherapy was tested in patients with locally advanced, unresectable, or metastatic sarcoma in a randomized multicenter, open-label phase II study (NCT02500797). In this study, only 5% of the patients receiving nivolumab responded to the treatment whereas addition of ipilimumab to nivolumab therapy led to an increased response rates highlighting a promising efficacy of the combination therapy [71].

In leiomyosarcomas patients, a phase II open label study is currently evaluating the effect of combination therapy with nivolumab and PARP inhibitor rucaparib (NCT04624178), and an interesting combination of nivolumab with trabectedin (macrophage affecting chemotherapeutic, see below) and oncolytic virus Talimogene Laherparepvec is to be evaluated in sarcoma patients in a currently recruiting phase II clinical trial (NCT03886311). Another phase I/II study in STSs patients aims to evaluate the efficacy and safety of nivolumab, together with ipilimumab and trabectedin as a first line treatment (NCT03138161). Nivolumab is further being evaluated in a combination therapy with chemotherapy (NCT04535713, NCT04339738), radiation therapy (NCT03307616), and targeted therapy (NCT03277924, NCT04416568).

Efficacy of ipilimumab therapy in STSs is being assessed in phase II clinical trials, including mostly combination therapies (Table 1). A single phase II clinical trial with ipilimumab monotherapy was carried out in patients with synovial sarcoma. However, the trial was terminated due to limited benefit of the treatment (NCT00140855).

ACT is a particular form of cell-based anticancer immunotherapy where both peripheral T cells and TILs can be used for ex vivo expansion and therapeutic administration to the patient [57]. This highly personalized therapy is to be studied in patients with advanced/metastatic STSs in a single center phase II open label study (NCT03725605). In this study, patients will be followed up for 15 months and the results are expected by 2023. In another phase II study, the efficacy of TIL infusion will be evaluated in a combination with chemotherapy in patients with multiple solid tumors, including STSs (NCT03935893). Appealing phase II study in synovial sarcoma patients is based on administering TBI-1301 (NY-ESO-1 specific TCR gene transduced autologous T lymphocytes) intravenously for 2 days following cyclophosphamide pre-treatment. Completion date is not yet estimated (NCT03250325).

A modification of traditional ACT is based on the infusion of the patients’ ex vivo expanded T cells after previous genetic modification of T-cells to express a chimeric antigen receptor (CAR) specific for a tumor antigen [72]. This therapy is called CAR T cell therapy [72]. To date, four phase II clinical trials have been initiated with the application of CAR T cells in STSs. All studies are currently recruiting (clinicaltrials.gov; July 1st 2021).

## 5. Regulatory T Cell (Treg) Infiltration

Regulatory T cells (Tregs, CD4^+^CD25^+^FOXP3-expressing T cells) regulate and suppress other cell types of the immune system. Their modulatory functions include production of cytokines, expression of inhibitory molecules, cytolytic functions, disruption of Ca2^+^ supply to the effector CD8^+^ lymphocytes, and multiple other mechanisms causing T cell anergy [73]. In the TME, Tregs were found to promote the tumor growth by restraining effective anti-tumor immune responses [73]. In STSs, a recent study in 192 surgically-treated STSs patients has revealed that the presence of Tregs is associated with the increased risk of local recurrence, irrespective of margins [74]. A study by D’Angelo et al. demonstrated that the proportions of FOXP3^+^ cells were relatively low as compared to CD4^+^ and CD8^+^ cells [51]. However, deep tumors were more likely to be associated with high FOXP3^+^ infiltration while superficial tumors had relatively low FOXP3^+^ infiltration [51]. A study by Klaver et al. further showed that pleomorphic sarcoma had a significantly lower fraction of Tregs as compared to leiomyosarcoma [48]. Moreover, Keung et al. observed that in patients with undifferentiated pleomorphic sarcoma undergoing neoadjuvant radiotherapy, tumors had an increased median number of Tregs which provided a rationale for a combination of radiotherapy and CPIs [75]. In a study by Que et al., both proportions of Tregs and the PD-L1 expression status were evaluated among 163 STSs patients [76]. In this study, a high load of Tregs positively correlated with the tumor stage, tumor grade and the depth of invasion. Also, PD-L1 expression, FOXP3^+^ Tregs infiltration and PD-L1/FOXP3 were significantly associated with overall survival in patients with undifferentiated pleomorphic sarcoma [76]. Since both PD-L1 and FOXP3 were highly expressed STS patients with poor prognosis, the authors call for combination of Treg depletion therapy and CPIs [76].

## 6. Targeting Tregs in Soft Tissue Sarcoma

The idea of blocking the immunosuppressive functions of Tregs has raised many concerns [77]. Principally, as Tregs ensure optimal immune responses to prevent autoimmunity, blocking their effector functions might result in a severe immune dysregulation [77].

Treg depletion for the purpose of enhancing antitumor immune responses has been previously tested mostly by an administration of Treg-specific cell-depleting antibodies. The target molecules included CD25, CTLA-4, GITR, 4-1BB, OX-40 and other molecules in diverse cancer types [77], Figure 2.

Molecule CD25 has a crucial role in the development and activation of T regs [73]. CD25 is a component of the high-affinity heterotrimeric IL-2 receptor that has been widely studied in the context of cancer [73]. Several issues, however, limit the wide use of anti-CD25 mAb [73]. Most importantly, effector T cells also share CD25 receptor making it difficult to selectively deplete Treg cells [78]. Therapeutic targeting of CD25 has been shown in metastatic melanoma patients to significantly deplete Tregs without impairing CD8^+^ T cell functions [78]. In STSs, a single clinical trial attempted to evaluate the efficacy of adoptive transfer with CD25 depleted autologous lymphocytes in rhabdomyosarcoma. Data are not yet available (NCT00923351).

Bempegaldesleukin is a recombinant form of human IL-2 that serves as a CD122-preferential IL-2 pathway agonist [79]. Hence, Bempegaldesleukin promotes proliferation of CD8^+^ T cells and NK cells without enhancing Treg activation [79]. A phase I/II study of bempegaldesleukin in combination with nivolumab has been initiated to evaluate the efficacy and safety in patients with recurrent or refractory malignancies, including rhabdomyosarcoma (NCT04730349).

CTLA-4 is constitutively expressed on T regs. A recent study by Zappasodi et al. has demonstrated that CTLA-4 blockade drives loss of Treg stability in glycolysis-low tumors [80]. Moreover, the anti-CTLA-4 therapy led to an increased IFN expression in the remaining Tregs and, therefore, attacked tumors at multiple levels [80]. Several clinical trials are currently ongoing with anti-CTLA-4 mAbs (see above). To date, clinical trials targeting different Treg molecules, such as GITR or OX-40 have not been initiated in STSs.

## 7. Natural Killer (NK) Cell Infiltration

NK cells belong to the family of innate lymphoid cells (ILC) and, in the context of cancer, NK cells are believed to be the main effector cells of the innate immune system [81]. NK cells are similarly to CD8^+^ T cells highly cytotoxic with the ability to produce proapoptotic Fas ligand and TRAIL, as well as the perforins and granzymes [81,82]. NK cells, are not only capable of triggering apoptosis in malignant cells but also shape the TME by a secretion of large amounts of cytokines [83]. Previous reports have also shown that NK cells prevent metastases by eliminating circulating tumor cells [84]. In STSs, the presence of NK cells has been reported through a limited number of studies. Sorbye et al. reported intratumoral NK cell status in 249 patients. In this study, a tendency towards improved overall survival was observed in STSs patients with high loads of CD57^+^ NK cells in the peritumoral area [85].

Bücklein et al. have provided a detailed analysis of NK cell presence and function in STSs patients and, unlike in other malignancies, peripheral blood NK cells exhibited a profound impairment in cell function, especially in the intermediate and high-grade STSs [86]. Judge et al. further demonstrated that intratumoral NK cells express more activation and exhaustion markers as compared to peripheral blood NK cells [87]. Furthermore, ex vivo stimulation with IL-15 further increased both activation and exhaustion markers in intratumoral and peripheral blood NK cells [87]. The authors observed a significant upregulation of TIGIT receptor and, therefore, suggested a therapeutic potential of TIGIT blockade together with IL-15 therapy [87].

## 8. NK Cell-Based Immunotherapies in Soft Tissue Sarcomas

IL-15 is a potent activator of NK cells with a major importance for NK cell proliferation and survival [88]. The advantages of IL-15 over IL-2 treatment include lower toxicity and lack of T-reg cell induction [89]. A phase I clinical trial evaluating the anti-tumor actions of IL-15 is administering autologous ex vivo activated NK cells with or without recombinant IL-15 to patients with solid tumors, including sarcomas (NCT01875601). Similarly, IL-15 is to be utilized as a compound of CAR T cells in a launching clinical trial with rhabdomyosarcoma and liposarcoma patients. In this trial, IL-15 is believed to deliver superior efficacy over classic CAR T cells. The genetic construct is called AGAR T cells (NCT04377932).

NK cells are known for their expression of a wide variety of activation and inhibitory receptors [90]. NKG2A is highly expressed on NK cells and transmits inhibitory signal [90], Figure 2. Anti-NKG2A antibody, monalizumab, is currently being tested among 13 different clinical trials, none of which is focused on STSs (clinicaltrials.gov, July 1st 2021).

Another NK receptor targeting agent is lirilumab, a pan-KIR2D blocker, which also has no ongoing clinical trials in STSs [91]. TIM-3 and Lag-3 are inhibitory checkpoint molecules with high expression in NK cells [92]. Although TIM-3 mAbs are evaluated in preclinical and phase-I clinical trials, data on their efficacy in STSs are lacking [93]. It should be noted that twelve anti-TIM-3 clinical trials have already been initiated (clinicaltrials.gov, July 1st 2021).TIGIT and CD96 are other NK cell receptors that serve as an optimal target for NK-mediated cancer immunotherapy [94]. While CD96 affects mainly the cytokine production of NK cells, TIGIT allows direct inhibition of NK cytotoxic functions through its ITIM domain [95]. Several clinical trials aimed at targeting TIGIT signaling pathway are currently underway, however, the efficacy of anti-TIGIT antibodies in STSs has not yet been evaluated [96]. Anti-CD96 therapy is still mostly under consideration in pre-clinical studies [94].

Adoptive cell transfer of NK cells has also become a promising treatment approach for advanced and/or metastatic diseases [97]. A phase II clinical trial in adult STSs evaluated the efficacy of cryosurgery in combination with NK cell immunotherapy. In this study, NK transfusions were given intravenously in three different time points with each infusion containing 8–10 billion cells (NCT02849366). Another pilot study aimed to evaluate the efficacy of intravenous infusion of expanded, activated haploidentical NK Cells (NCT02409576). NK cells were administered together with chemotherapy, radiotherapy, and cytokine support with IL-2 cytokine. A study combining NK cell infusions together with ALT-803, which is a pharmacological IL-15 superagonist is based on NK cells from non-HLA matched donors (NCT02890758). Even though, allogenic NK cell infusions generally showed poor anti-tumor activity in clinical trials, the combination with ALT-803 could significantly enhance the NK cell cytotoxicity and finally provide desirable responses in STSs [98].

## 9. Macrophages

Macrophages differentiated from circulating monocytes that efficiently migrate within the tissue and continue trafficking into the TME are called tumor-associated macrophages (TAMs) [99]. TAMs are capable of phagocytosis, as well as regulation of the tissue growth and repair [99]. With the ability to produce immunosuppressive cytokines, such as IL-10 and TGF-β, to upregulate Tregs and to promote Th2 immune response, TAMs have become major contributors to the cancer progression [100]. However, it is the TME and the tumor-derived signals that intensify TAM functions [100].

In different cancer types, TAMs were associated with poor prognosis and reduced overall survival (OS) [101]. Even though TAMs mainly exploit protumorigenic mechanisms, several antitumor functions of TAMs have also been reported [102].

In a recent study by Dancsok et al., TAMs were investigated in 1242 sarcoma specimens [103]. The study revealed that across nearly all sarcoma types, macrophages outnumber TILs and thus dominate the immune landscape of STSs [103]. In this study, TAMs were most frequently observed in pleomorphic sarcomas, such as undifferentiated pleomorphic sarcoma and dedifferentiated liposarcomas [103]. As opposed, the lowest macrophage infiltration was found in synovial sarcoma, myxoid liposarcoma, clear cell sarcoma, and low-grade fibromyxoid sarcoma [103]. In synovial sarcoma, another study also reported that lower TAM infiltration is associated with better overall survival [104]. A recent study by Tsagozis et al. has highlighted that the immune cells infiltrating STSs are poorly characterized to date [105]. Therefore, the authors provided an immunohistochemical analysis of STSs tumors with different histology and, in accordance with the study by Dancsok et al., also found much higher densities of TAMs as compared to TILs in the sarcoma TME [103,105]. In this study, the pan-macrophage marker CD68 correlated with high immune cell infiltration in general and most macrophages were M2-polarized [105]. In a study by Shailaja et al., specific alteration in TAM densities were observed in STSs patients responding to neoadjuvant chemotherapy, suggesting that chemotherapy does indeed significantly modulate the TME of STSs [106].

In leiomyosarcoma, authors Lee et al. described a significant association between high density of TAMs and worse disease-specific survival [107]. This was further supported by another study correlating the high load of TAMs with poor survival in leiomyosarcomas [108]. Undifferentiated pleomorphic sarcoma was already reported to have high levels of macrophage infiltration [103]. A study by Shiraishi et al. further revealed that a high percentage of TAMs is related to shortened overall survival and to high AJCC stage and high FNCLCC grade [109]. This is particularly important since tumor grade is the most important prognostic factor for STSs to date [9]. Similar to undifferentiated pleomorphic sarcoma and leiomyosarcoma, myxoid liposarcoma was also shown to be infiltrated with M2 macrophages that negatively determine the patients’ outcome and thus, remain a candidate for macrophage-targeted therapies [103,110].

## 10. Therapies Targeting Macrophages

Since macrophages represent the predominant cell type in most STSs, different clinical trials have been initiated to either suppress the pro-tumorigenic functions of TAMs or to modulate the natural ability of TAMs to eliminate target cells [111].

CD47 is a transmembrane protein that is highly expressed on neoplastically transformed cells [112]. CD47 binds to SIRPα, and this receptor-ligand interaction releases “don’t eat me” signals that inhibit macrophage-mediated phagocytosis (Figure 2) [112]. Despite promising pre-clinical studies with anti-CD47 mAbs promoting the macrophage-mediated phagocytosis towards malignant cells of leiomyosarcoma, clinical trials in human STSs have not yet been carried out [113]. A phase I clinical trial administering an antibody-drug conjugate SGN-CD47M in patients with various solid tumors, including STSs, was terminated in 2020 due to the sponsors’ decision (NCT03957096).

A multikinase inhibitor Pexidartinib (PLX3397) is currently being evaluated in a phase I/II clinical trial for the treatment of multiple sarcomas including liposarcoma, leiomyosarcoma, synovial sarcoma, and rhabdomyosarcoma (NCT02584647). Pexidartinib is an inhibitor of proto-oncogene receptor tyrosine kinase (KIT), colony-stimulating factor-1 receptor (CSF1R) and FMS-like tyrosine kinase 3 (FLT3), and was shown to decrease the load of intratumoral TAMs and increase the proportions of CD4^+^ and CD8^+^ T cells, thus contributing to the tumor regression [114]. Due to diverse therapeutic targets of pexidartinib, its role in targeting macrophages include limiting their differentiation, extravasation, and polarization towards M2 phenotype [114,115]. Results of the first clinical trials in sarcoma tumors are eagerly awaited.

Trabectedin is a chemotherapeutic drug which was recently approved in the treatment of advanced STSs [116]. Trabectedin is classified as an alkylating drug, as it interferes with the cell division and the DNA repair. However, previous studies have also highlighted the ability of trabectedin to deplete both circulating monocytes and TAMs [117]. The selectivity of trabectedin against mononuclear phagocytes was explained as a result of rapid activation of caspase 8 by membrane signaling TRAIL receptors which are highly expressed in monocytes/macrophages [117]. Since most STSs are primary chemotherapy resistant, it may be the macrophage depletion that lies behind the efficacy of trabectedin in STSs [117,118].

Other possible therapeutic targets include chemokine and chemokine receptors regulating the macrophage trafficking towards the tumor [119]. CCL2-CCR2 axis serves as the major macrophage chemoattractant and has been proposed a suitable therapeutic target by previous studies [120]. Clinical trials with chemokine blockade in STSs are lacking but urgently needed to open novel options in the treatment of STSs [120].

## 11. Discussion

Soft tissue sarcomas are rare but mostly lethal mesenchymal tumors [4]. STSs are extremely biologically and clinically diverse tumors with more than 80 histological subtypes [2,3]. Due to such rarity of these tumors, clinical trials often evaluate all STSs types together, which may contribute to the mixed results from early immunotherapy clinical trials [103,121]. Localized STSs can be effectively treated by surgery with a five-year relative survival rate of 81% (STS statistics, Cancer.net, ASCO). However, the treatment of metastatic STSs has not changed for decades and, with the current conventional therapies, only small chances for the improvement in the overall survival rates are secured [4]. The TME of STSs tumors is infiltrated by diverse proportions of immune cells which provides a rationale to stratify patient according to the TME immune parameters [4,48]. CD8^+^ T cells belong to the main antitumor players in the TME, the infiltration of the TME with CD8^+^ T cells, and the expression of immune checkpoint molecules have become prerequisites for an effective immunotherapy with CPIs [122]. Pleomorphic sarcoma and myxofibrosarcoma were shown to have one of the highest infiltration with CD8^+^ T cells and the highest expression of PD-1 [48]. Therefore, these tumors are expected to equally benefit from CPIs [48]. In addition, pleomorphic sarcoma displayed a highly comparable immune landscape to malignant melanoma, suggesting an optimal suitability for immunotherapeutic approaches [48]. Interestingly, in larger tumors, CD8^+^ T cells tended to become excluded from the TME which could imply a limited efficacy of CPIs in tumors of greater size [58]. Leiomyosarcomas and liposarcomas have generally low CD8^+^ T cell infiltration among different studies which is reflected in the greatly disappointing results in clinical trials [71]. Out of T cell immunotherapies, CPIs are the most widely used agents. However, both nivolumab and ipilimumab in monotherapies showed only limited efficacy [71,123]. The undifferentiated pleomorphic sarcoma histology is associated with better response and CPIs combination therapies, such as nivolumab plus pembrolizumab, significantly increase the response rates in STSs patients. Other promising clinical trials include the ACT or CAR T cell therapy [124].

Poor immunotherapy responses in patients with leiomyosarcomas may also be associated with their relatively high loads of Tregs as compared to pleomorphic sarcomas [48]. TME infiltration with Tregs positively correlates with the tumor grade, stage, and the depth of invasion [76]. Moreover, in undifferentiated pleomorphic sarcomas, Treg infiltration increases after neoadjuvant radiotherapy and affects the overall survival of patients [76]. Blocking the immunosuppressive functions of Tregs, however, raises many safety concerns and, to date, mostly anti-CTLA-4 agents show a great deal of promise in affecting both CD8^+^ T cells in addition to Tregs [77,80]. Data regarding the presence and phenotypes of NK cells in STSs are quite limited. Hence, detailed analyses would be truly beneficial in STSs. While intratumoral NK cells of STSs exhibit numerous activation and exhaustion markers, peripheral NK cells in STS patients were observed as functionally impaired [86,87]. Patients undergoing NK-cell based immunotherapies mostly profit from administration of IL-15, a potent NK cell activator, while anti-NKG2A therapy is still lacking among clinical trials in STSs [125,126]. Macrophages dominate the immune landscape of STSs and are associated with the tumor grade and outnumber TILs across nearly all sarcoma types [103]. TAMs are frequently observed in pleiomorphic sarcomas, while the infiltration among liposarcomas, clear cell sarcomas, and synovial sarcomas is relatively low. It should be noted that alterations in TAM densities were shown in patients after neoadjuvant chemotherapy [106]. Therapies targeting macrophages are thus very promising in STSs. Preclinical studies reveal the potential of anti-CD47 therapy for leiomyosarcoma, and clinical trials employ mostly pexidarinib or trabectedin [103,120].

With the current knowledge of diverse immune cell populations infiltrating STS tumors, the ongoing clinical practice could fundamentally change and allow stratification of patients based on the immune landscape of the TME. As distinct histotypes of STSs are recognized to be infiltrated with immune cells in diverse proportions and are only sensitive to specific cytotoxic drugs, it is likely that also preclinical research will focus on deciphering the optimal histology-driven therapeutic regimens.

It is already clear that clinical trials aiming at multiple immune cell populations, such as T cells and macrophages that are triggering both the innate and adaptive immunity, bring a great deal of promise. As shown in clinical trials, immunotherapies can be easily combined and chemotherapy can serve as a powerful tool to sensitize TME to immunotherapy in these mostly chemoresistant tumors.

## Figures and Tables

**Table 1:** A list of phase II and III active or recruiting clinical trials with nivolumab, pembrolizumab, and ipilimumab in soft tissue sarcomas. Excluded were withdrawn clinical trials, completed trials and trials of unknown status. Red triangle indicates phase III clinical trials.

Phase II + III Clinical Trials						
Drug Name	Diagnosis	Trial Design	Setting	CPI Dosage Regimen	Estimated Study Completion	Identifier
Nivolumab	Soft tissue sarcoma	Non-randomized	Combination therapy: Ipilimumab, Cryoablation	3 mg/kg every 3 weeks x 4 doses.	October 2025	NCT04118166
Nivolumab	Soft tissue sarcoma	Randomized	Combination therapy: Relatlimab	240 mg every 2 weeks	September 2024	NCT04095208
Nivolumab	Soft tissue sarcoma	Non-randomized	Combination therapy: Trabectedin	240 mg every 3 weeks	October 2022	NCT03590210
Nivolumab	Soft tissue sarcoma	Randomized	Combination: Cabozantinib, Ipilimumab	3 mg/kg every 3 weeks x 4 doses, followed by 480 mg every 4 weeks	January 2027	NCT04551430
Nivolumab	Sarcoma, Desmoid, Chondroma	Non-randomized	Combination: Trabectedin, Talimogene Laherparepvec	240 mg every 2 weeks	December 2022	NCT03886311
Nivolumab	Advanced/metastatic sarcoma	Non-randomized	Combination: NKTR-214	360 mg every 3 weeks	September 2023	NCT03282344
Nivolumab	Advanced/metastatic sarcoma	Non-randomized	Combination: Trabectedin, Ipilimumab	3 mg/kg every 2 weeks up to 26 doses	March 2022	NCT03138161
Nivolumab	Advanced/metastatic sarcoma	Non-randomized	Combination: Gemcitabine, Doxorubicin, Docetaxel	240 mg IV on Day 1 of each cycle	December 2025	NCT04535713
Nivolumab	Soft tissue sarcoma	Non-randomized	Combination: Sunitinib	240 mg every 2 weeks	September 2022	NCT03277924
Nivolumab	Recurrent/refractory sarcoma	Non-randomized	Monotherapy	240 mg every 2 weeks	March 2029	NCT03465592
Nivolumab	Resectable or recurrent dedifferentiated/undifferentiated pleomorfic sarcoma	Randomized	A: monotherapy; B combination with Ipilimumab; C combination with RT; D combination with Ipilimumab and RT	IV on days 1, 15 and 29 in A, B; IV over 1 h on days 1, 15, 29 and 43 in C, D	October 2021	NCT03307616
Nivolumab	Angiosarcoma	Randomized	A: nivolumab, paclitaxel; B paclitaxel; C: nivolumab, cabozantinib S-malate	I.V. on Day 1 of each cycle, cycles repeat every 4 weeks	September 2023	NCT04339738
Nivolumab	Soft tissue sarcoma	Non-randomized	Combination: AL3818	240 mg every 2 weeks	December 2022	NCT04165330
Nivolumab	Epitheloid sarcoma	Non-randomized	Combination: Ipilimumab	Nivo and Ipi at predetermined dosage day 1 of a 21-day cycle for 4 cycles.	October 2025	NCT04416568
Nivolumab	Uterine sarcomas	Non-randomized	Monotherapy	480 mg IV once every 4 weeks	August 2022	NCT03241745
Nivolumab	Soft tissue sarcoma	Non-randomized	Combination: BA3011	Unspecified	January 2022	NCT03425279
Nivolumab	Sarcoma 2nd-line and relapsed/refractory	Non-randomized	Combination: NKTR-262, bempegaldesleukin	360 mg every 3 weeks	December 2021	NCT03435640
Nivolumab	Leiomyosarcoma	Non-randomized	Combination: Rucaparib	480 mg i.v. on day 1 of every four-week cycle	November 2022	NCT04624178
Nivolumab	Angiosarcoma, endometrial carcinosarcoma	Non-randomized	Monotherapy, Combination: Ipilimumab	Unspecified	August 2021	NCT02834013
Nivolumab	Advanced/metastatic sarcoma	Randomized	Combination: Ipilimumab	Nivolumab: 3 mg/kg i.v. every 2 weeks for 4 cycles; Ipilimumab 1 mg/kg IV over 60 min every 6 weeks for 4 cycles	August 2025	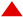 NCT04741438
Pembrolizumab	Advanced sarcoma	Non-randomized	Combination: Lenvatinib	200 mg as a 30-min IV infusion, Q3W +/−3 days	March 2024	NCT04784247
Pembrolizumab	Advanced sarcoma	Non-randomized	Combination: Metronomic Cyclophosphamide	200 mg every 3 weeks on day 8 for 3 weeks	August 2021	NCT02406781
Pembrolizumab	Soft tissue sarcoma of the Extremity	Randomized	Combination: Radiotherapy	200 mg i.v. every 3 weeks	July 2025	NCT03092323
Pembrolizumab	Soft tissue sarcoma	Non-randomized	Combination: Axitinib	200 mg i.v. infusion every 21 weeks, max up to 2 years	December 2022	NCT02636725
Pembrolizumab	Advanced/metastatic sarcoma	Non-randomized	Combination: Epacadostat	200 mg/dose Day 1, Q 3 weeks	January 2022	NCT03414229
Pembrolizumab	Soft tissue sarcoma	Non-randomized	Combination: Eribulin	Pembrolizumab every 3 weeks	August 2024	NCT03899805
Pembrolizumab	Advanced/metastatic soft tissue sarcoma	Non-randomized	Combination: Doxorubicin	200 mg intravenously every 3 weeks	February 2025	NCT03056001
Pembrolizumab	Soft tissue sarcoma	Non-randomized	Combination: Radiotherapy	i.v. every 3 weeks for 3 months	June 2023	NCT03338959
Pembrolizumab	Advanced/metastatic sarcoma	Non-randomized	Combination: Alimogene Laherparepvec (T-VEC)	Every 3 weeks	March 2022	NCT03069378
Pembrolizumab	Sarcoma of extremities	Non-randomized	Combination: Isolated Limb infusion (ILI)	200 mg i.v. every 3 weeks	April 2023	NCT04332874
Pembrolizumab	Leiomyosarcoma and Undifferentiated Pleomorphic Sarcoma	Non-randomized	Combination: Gemcitabine	200 mg every 3 weeks	December 2020	NCT03123276
Pembrolizumab	Advanced/metastatic Synovial Sarcoma	Non-randomized	Combination: Interferon gamma-1b	200 mg i.v. every 3 weeks	April 2022	NCT03063632
Pembrolizumab	Soft tissue sarcoma	Non-randomized	Combination: Intra-tumoral BT-001	200 mg intravenously every 3 weeks	November 2024	NCT04725331
Pembrolizumab	Sarcoma	Non-randomized	Monotherapy	200 mg i.v. every 3 weeks	December 2023	NCT03012620
Ipilimumab	Undifferentiated Pleomorphic Sarcoma Or Myxofibrosarcoma	Randomized	Combination: Envafolimab	1 mg/kg every 3 weeks for a total of 4 doses	July 2022	NCT04480502
Ipilimumab	Soft tissue sarcoma	Non-randomized	Combination: Aldesleukin, nivolumab, fludarabine, cyclophosphamide	Unspecified	June 2024	NCT03449108
Ipilimumab	Sarcoma	Non-randomized	Combination: INT230-6	Day 1 every 3 weeks for four treatments	July 2022	NCT03058289

## Data Availability

Data sharing is not applicable to this article.
